# MSC-Derived Extracellular Vesicles Activate Mitophagy to Alleviate Renal Ischemia/Reperfusion Injury via the miR-223-3p/NLRP3 Axis

**DOI:** 10.1155/2022/6852661

**Published:** 2022-05-20

**Authors:** Zejia Sun, Zihao Gao, Jiyue Wu, Xiang Zheng, Shizhao Jing, Wei Wang

**Affiliations:** Department of Urology, Capital Medical University Beijing Chaoyang Hospital, Beijing 100020, China

## Abstract

**Background:**

MSC-derived extracellular vehicles (EVs) exhibit a protective functional role in renal ischemia/reperfusion injury (RIRI). Recent studies have revealed that mitophagy could be a potential target process in the treatment of RIRI. However, whether MSC-derived EVs are involved in the regulation of mitophagy in RIRI remains largely unknown to date.

**Methods:**

RIRI model was established *in vivo* in mice by subjecting them to renal ischemia/reperfusion. TCMK-1 cells were subjected to hypoxia/reoxygenation (H/R) stimulation to mimic RIRI *in vitro*. BMSCs and BMSC-derived EVs were isolated and identified. Renal injury was assessed using H&E staining. The qPCR and western blot analyses were conducted to detect the mRNA and protein levels. Apoptosis was evaluated using the TUNEL assay and flow cytometry analysis. The EVs, autophagosomes, and mitochondria were observed using TEM. The colocalization of autophagosomes with mitochondria was confirmed through the confocal assay. The direct binding of miR-223-3p to NLRP3 was validated through the dual-luciferase assay.

**Results:**

BMSCs and BMSC-derived EVs were successfully isolated from mice and identified. The protective effect of BMSC-derived EVs against RIRI was validated both *in vitro* and *in vivo*, which was indicated by a decrease in apoptosis and inflammasome activation and an increase in mitophagy. However, this protective effect was impaired in the miR-223-3p-depleted EVs, suggesting that miR-223-3p mediated this protective effect. Further mechanistic investigation revealed that miR-223-3p suppressed inflammasome activation to enhance mitophagy by directly targeting NLRP3.

**Conclusion:**

In conclusion, the protective role of BMSC-derived EVs and exosome-delivered miR-223-3p in RIRI was validated. Exogenous miR-223-3p directly targeted NLRP3 to attenuate inflammasome activation, thereby promoting mitophagy.

## 1. Introduction

Acute kidney injury (AKI) is a common clinical syndrome with rapid development and a high incidence of renal impairment, which leads to chronic kidney disease and end-stage renal disease [1]. The main cause of AKI is renal ischemia/reperfusion injury (RIRI), which is characterized by limiting the blood supply to the kidneys, followed by restoring the blood flow and reoxygenation. Several possible mechanisms may underlie RIRI, including calcium overload, damage caused by free radicals, the interaction between vascular endothelial cells and neutrophils, and angiotensin II [2]. Clinically, RIRI is caused by acute injuries, such as those during kidney transplantation, iatrogenic injury, and hemorrhagic shock, which leads to severe tissue or organ damage [3]. Currently, no effective drugs are available to protect the kidney from ischemia/reperfusion injury. Therefore, for early clinical diagnosis and effective treatment of RIRI, it is important to explore the mechanisms underlying the renal ischemia/reperfusion injury.

Mitochondria serve as essential energy-generating components in tissue homeostasis and a channel for programmed apoptosis and necrosis. Therefore, the quality and quantity of mitochondria are strictly controlled in a living cell. The quality of mitochondria is maintained through mitophagy, which is an evolutionarily conserved process involving the autophagic phagocytosis and destruction of mitochondria [4, 5]. Studies conducted in recent years have reported that mitophagy mediates several cellular processes and diseases, including IRI. In IRI pathology, mitophagy is generally activated to exert a protective role [6]. Tang et al. have revealed that BNIP3-mediated mitophagy protected the kidney against IRI [7]. Moreover, HIF-1*α* is reported to ameliorate RIRI via the activation of BNIP-mediated mitophagy [8]. Studies have also validated that in cerebral ischemia/reperfusion injury, mitophagy is promoted by FUNDC1 to protect the neurons from IRI [9]. Furthermore, in cardiac ischemia/reperfusion injury, melatonin was observed to play a protective role via mitophagy activation. These pieces of evidence suggest that regulating mitophagy could serve as a potent approach for the treatment of ischemia/reperfusion injury. Therefore, in the present study, the regulatory mechanism of mitophagy was further explored.

Mesenchymal stem cells (MSCs) are a type of pluripotent stem cells exhibiting the characteristics of self-renewal and multidirectional differentiation [10]. MSCs are present in the bone marrow, skeletal muscles, adventitia, and trabeculae. Currently, MSCs are among the most widely used cells in the clinical treatment of various diseases [11]. MSC-derived extracellular vesicles with diameters ranging from 40 nm to 100 nm are defined as exosomes and are secreted by the MSCs to mediate the function of these cells [12]. Several studies have unveiled the protective role of MSCs and MSC-derived exosomes in IRI [13, 14]. For instance, a study by Lai et al. revealed that MSC-derived exosomes attenuated the myocardial ischemia/reperfusion injury [15]. Zhao et al. reported that MSC-derived exosomes delivered miR-182 to regulate macrophage polarization in myocardial ischemia/reperfusion injury [16]. In renal ischemia/reperfusion injury, melatonin is reported to elevate the protective potential of MSC-derived exosomes [17]. Moreover, the C-C motif chemokine receptor-2- (CCR2-) positive exosomes from MSCs are reported to alleviate the renal ischemia/reperfusion injury by regulating the function of macrophages [18]. These reports suggest that MSC-derived EVs including exosomes play a protective functional role in RIRI. However, the underlying mechanism remains largely unknown to date and requires further investigation to be elucidated.

The microRNAs (miRNAs) are a group of short noncoding RNAs with 18–25 nucleotides. Several miRNAs are verified to be dysregulated in the ischemia/reperfusion condition and reported to exhibit functional roles in the progress of ischemia/reperfusion [19, 20]. Interestingly, as a component in EVs, miRNAs have been demonstrated to be involved in the protective role of EVs in IRI. It has been verified that miR-124 from M2 microglia-derived EVs attenuated cerebral IRI [21]. Moreover, miR-181 delivered from the MSC-derived exosomes was confirmed to ameliorate myocardial IRI [22]. A study by Qin et al. revealed that miR-223-3p suppressed the necroptosis in myocardial ischemia/reperfusion [23]. In addition, miR-223-3p is reported to participate in the functional role of exosomes in cerebral ischemia/reperfusion via the suppression of M1 polarization [24]. However, whether miR-223-3p exhibits a similar functional role in renal ischemia/reperfusion remains largely unknown to date.

In the present study, the protective role of miR-223-3p from the MSC-derived EVs with a mean diameter of ~70 nm in RIRI was validated. Exogenous miR-223-3p promoted mitophagy and suppressed inflammasome activation by directly targeting NLRP3. The findings of the present study would contribute to understanding the protective regulatory role of EVs in RIRI.

## 2. Materials and Methods

### 2.1. Cell Culture and Hypoxia/Reoxygenation (H/R) Model

The mouse tubular epithelial cell line TCMK-1 was purchased from the National Collection of Authenticated Cell Cultures. The TCMK-1 cells were cultured in a medium containing 89% DMEM (Invitrogen, USA), 10% FBS (Gibco, USA), and 1% Penicillin–Streptomycin solution (Sigma, USA) at 37°C under a 5% CO_2_ atmosphere. The H/R model was established using 94% N_2_, 5% CO_2_, and 1% O_2_ treatments for 12 h, followed by incubating the TCMK-1 cells in a normal atmosphere for another 12 h.

### 2.2. Isolation and Identification of the Mouse Bone Mesenchymal Stem Cells (BMSCs)

The mice for the experiments (male C57BL/6 mice, 6–8-week-old, 20–25 g in body weight) were procured from Beijing MaiDeKangNa Biotechnology Company. All mice were raised under appropriate conditions with a 12-h light/12-h dark photoperiod and free access to water and chow. The animal experiment protocols were approved by the Capital Medical University of the Beijing Chaoyang Hospital.

In order to obtain the tissues for analysis, the C57BL/6 mice were subjected to cervical dislocation followed by dipping in 75% alcohol for 10 min. The bilateral thigh skin was excised to remove the femoral bone and tibia. Subsequently, the epiphyseal ends on both sides of the removed bones were excised under sterile conditions, and the bone marrow was washed out by pumping Dulbecco's minimal essential medium (Invitrogen) into the bone marrow cavity. The obtained cell suspension was centrifuged at 1500 g for 5 min and then resuspended in a modified medium comprising 89% DMEM, 10% FBS, and 1% Penicillin–Streptomycin solution.

Cells from the third passage were collected, and cell density was adjusted to 1 × 10^7^ cells/mL. Flow cytometry was employed to identify the surface markers of MSCs using the CD45 Monoclonal Antibody (12-0451-82, ThermoFisher, USA), CD73 Monoclonal Antibody (12-0739-42, ThermoFisher), and CD105 Monoclonal Antibody (12-1051-82, ThermoFisher).

### 2.3. EV Isolation and Particle Analysis

BMSCs were cultured in exosome-depleted FBS (Catalog number: A2720801, Gibco) medium EV isolation. The culture medium (a total volume of 740 ml) containing primary BMSCs (10^6^–10^7^ cells in a 10 cm dish) was centrifuged at 2000 g for 30 min. The resulting supernatant was transferred to a fresh empty tube and subjected to exosome extraction using the Total Exosome Isolation kit (Catalog number: 4478359, Invitrogen). The reagents of the kit were added to the cell-free culture medium, the mixture was vortexed until a homogenous solution was obtained, and the samples were then incubated overnight at 4°C. Afterward, the supernatant was discarded, and the EVs were collected in the form of pellet settled at the bottom of the tube. The pellet was resuspended and subjected to EV morphology was observed by using transmission electron microscopy (TEM, HT-7700, Hitachi) and particle size and concentration were determined by qNano GOLD instrument (IZON, New Zealand). The marker proteins CD63 and CD9 were detected using western blot analysis.

### 2.4. Sample Preparation for TEM

The structures of the mitochondria, autophagosomes, and lysosomes were visualized using TEM. Approximately 1 mm^3^-sized renal tissues were fixed using glutaraldehyde and osmium acid treatments for 2 h. The fixed samples were dehydrated using a gradient of alcohol. The dehydrated samples were embedded using anhydrous acetone and the embedding agent and then excised into ultrathin sections. Uranyl acetate and lead citrate were prepared for the staining of these sections. The stained sections were finally visualized under a transmission electron microscope.

### 2.5. Transfection

The miR-RNA was synthesized by Sangon Biotech (Shanghai, China). The primary BMSCs or TCMK-1 cells were transfected with 50 nmol/L of the miR-RNA or an equal volume of negative control using Lipofectamine 2000 reagent (Invitrogen). The transfection efficiency was determined by performing qPCR.

### 2.6. Establishment of the Renal Ischemia/Reperfusion (I/R) Model and Exosome Injection

In order to establish the I/R model, C57BL/6 mice were first subjected to fasting for 12 h and then anesthetized using isoflurane for surgery. The back hair of the anesthetized mouse was shaved and disinfected using a 75% alcohol wipe. Afterward, the skin and the muscles close to the spine were removed to expose the renal tissue. The ischemia procedure was implemented by occlusion of the renal pedicles on both sides using a clamp for 30 min. The mice were immediately supplemented with normal saline. After 30 min, the clamps were removed, and the mice were subjected to 24 h of reperfusion. The mice were then euthanized using an intravenous overdose of isopentarbital. The whole blood and the kidney tissue of the mice were collected for subsequent examinations. The mice were randomly categorized into groups as described below (*n* = 6 in each group).

The control group mice did not receive any treatment. In the sham group, the skin and the muscles were removed while ischemia and reperfusion were not performed. In the I/R+EXO group, after 1 h of reperfusion, 5 × 10^10^ EVs in 100 *μ*L PBS were injected into the mouse via the caudal vein. In the H/R+EXO (inhibitor NC/miR-223-3p inhibitor) group, the EVs injected in mice were extracted from the BMSCs transfected with inhibitor NC or the miR-223-3p inhibitor.

### 2.7. Exosome Internalization

The EVs from BMSCs were labeled with PKH67 (Merk, German) and then subjected to ultracentrifugation to remove the excess stain. After washing the TCMK-1 cells with PBS, 1 mL of medium containing 5 × 10^10^ EVs with green fluorescence was added to these cells. After 24 h of incubation, DAPI treatment was performed for staining the cell nuclei. Exosome internalization was visualized under a fluorescence microscope.

### 2.8. Analysis of mtDNA Damage Using qPCR

The integrity of the mitochondrial DNA (mtDNA) was evaluated using the mtDNA Isolation kit (BioVision) in accordance with the manufacturer's instructions. Briefly, 5 × 10^7^ cells were resuspended in the cytosol extraction buffer, followed by homogenization for 10 min and then centrifugation at 1200 g and 4°C for 10 min. The resulting supernatant was centrifuged again at 4°C for 30 min. The supernatant was discarded this time, and the pellet settled at the bottom was resuspended in the cytosol extraction buffer. After centrifuging the pellet suspension at 10000 g and 4°C for 30 min, the mitochondria were isolated and collected in the precipitate. The mitochondria-containing precipitate was suspended in the lysis buffer, and Enzyme B Mix was added to this mixture followed by incubation at 50°C in a water bath for 60 min until the solution became clear. The pellet obtained was washed using ethanol to obtain the mtDNA for the subsequent PCR analysis.

### 2.9. Quantitative Real-Time PCR (qRT-PCR)

Total RNA was isolated using the Takara RNA kit (Japan) in accordance with the manufacturer's instruction. In order to synthesize cDNA from this isolated total RNA, 2 *μ*L gDNA Eraser Buffer, 1 *μ*L gDNA Eraser, 5 *μ*L RNase-free dH_2_O, and 1 *μ*L RNA were mixed and incubated at 42°C for 2 min. Subsequently, 4 *μ*L PrimerScript Buffer, 1 *μ*L PrimerScript RT Enzyme Mix I, 1 *μ*L RT Primer Mix, and 4 *μ*L RNase-Free dH_2_O were added to this mixture followed by incubation at 37°C for 15 min. The reagents for qRT-PCR were prepared as described in the kit instructions. The PCR was performed under the following reaction conditions: 95°C for 30 s; 40 cycles of 95°C for 5 s and then 60°C for 34 s. The expression of miR-223-3p was determined using the 2^-*ΔΔ*CT^ method. The primer sequences used for miR-223-3p were as follows: Forward: 5′-GAA GCT GTA CCT AAC ATA CCG TG-3′; Reverse: 5′-GAT TGG TCG TGG ACG TGT CG-3′.

### 2.10. Western Blot Analysis

The RIPA lysis buffer was mixed with phenylmethylsulfonyl fluoride (Cell signaling technology, CST, USA), and the resulting mixture was used for protein extraction. The collected protein samples were separated on a 10% SDS-PAGE gel and then transferred to PVDF membranes. After blocking the unspecific sites in the PVDF membranes, these membranes were incubated overnight with the following primary antitetraspanins IgG antibodies: anti-Calnexin (Abcam, ab22595, 1 : 1000), anti-CD9 (Abcam, ab223052, 1 : 1000), anti-CD63 (Abcam, ab217345, 1 : 1000), anti-PINK1 (Abcam, ab23707, 1 : 1000), anti-Parkin (Invitrogen, 13399, 1 : 1000), anti-LC3 (Abcam, ab192890, 1 : 1000), anti-TOMM20 (Invitrogen, PA5-52843, 1 : 1000), anti-TIMM23 (Invitrogen, PA5-98194, 1 : 1000), anti-NLRP3 (Abcam, ab263899, 1 : 1000), anti-ASC1 (Invitrogen, PA5-90403, 1 : 1000), anti-cleaved caspase1 (CST, #89332, 1 : 1000), anti-cleaved caspase 3 (CST, #9661S, 1 : 1000), anti-Bcl-2 (CST, #3498, 1 : 1000), and anti-BAX (CST, #14796S, 1 : 1000). Afterward, the membranes were incubated with secondary antimouse IgG or antirabbit IgG antibodies (CST, 1 : 10000) for 2 h. After washing the membranes, the protein bands were visualized, and the protein expression levels were quantified relative to the *β*-actin levels.

### 2.11. H&E Staining

The kidney tissue was soaked in 10% formalin and then dehydrated and hyalinized using alcohol and xylene, respectively. Subsequently, the tissue was embedded in paraffin and then sliced into thin sections of 5–8 *μ*m. After dewaxing the excised sections using xylene and alcohol, the sections were subjected to hematoxylin and eosin staining.

### 2.12. Blood Urea Nitrogen (BUN) and Serum Creatinine (sCr) Examinations

The creatinine assay kit (Abcam) and urea assay kit (Abcam) were used for the detection of sCr and BUN, respectively. The serum was separated from the whole blood samples and subjected to the detection of BUN and sCr contents conducted in accordance with the manufacturer's instructions. The BUN or sCr content in the evaluated samples was calculated according to the calculation method given.

### 2.13. TUNEL Assay

TUNEL assay (BOSTER, China) was performed to evaluate the level of apoptosis in the renal tissue samples. First, the tissue sections were digested using protease and then incubated with a labeling buffer containing TDT and Bio-D-UTP at room temperature for 2 h. After washing the sections with TBS, the blocking solution was added, followed by 30 min of incubation at room temperature. Afterward, diluted SABC-AP was added to these sections, followed by 60 min of incubation and then three rinses with TBS. Finally, BCIP/NBT was added to the sample, followed by 30 min of incubation in the dark. Apoptotic cells were observed under a fluorescence microscope.

### 2.14. Annexin V/PI Assay for Apoptosis

Cell apoptosis was evaluated using the Annexin V/PI kit (Sigma Aldrich). The prepared cells were resuspended in the binding buffer at the density of 10^6^ cells per mL. Next, 5 *μ*L of Annexin V-FITC and 10 *μ*L of PI solution were added to the cell suspension, followed by incubation for 15 min in the dark. The samples were examined using flow cytometry within 1 h of staining.

### 2.15. Confocal Microscopy Observation of Mitochondria and Autophagosomes

In order to observe the colocalization of mitochondria and autophagosomes, the TCMK-1 cells were cotransfected with GFP-LC3B and DsRed-Mito plasmids (Gene Pharm). After 48 h, the overlap of green and red fluorescence emission was visualized under a confocal microscope.

### 2.16. Dual-Luciferase Assay

The binding sequences of miR-223-3p and NLRP3 were predicted using TargetScan. The full-length binding site sequences of the NLRP3 3′-UTR were amplified and cloned into the pGL3 vector to generate the plasmids pGL3-WT-NLRP3 and pGL3-MUT-NLRP3 (Gene Pharm). Subsequently, the BMSCs or TCMK-1 cells were cotransfected with the luciferase reporter, and the miR-223-3P mimic or mimic NC using Lipofectamine 2000 reagent. After 48 h, the luciferase activity was detected using the Dual-Lucy Assay kit (ThermoFisher Scientific) in accordance with the manufacturer's protocol.

### 2.17. Statistical Analysis

All data were obtained from a minimum of three repeated experiments and were presented as mean ± SD. The data were analyzed using the SPSS 20.0 software. Student's *t*-test or one-way analysis of variance was used for analyzing the differences. The threshold of statistical significance was set at a *P* value < 0.05.

## 3. Results

### 3.1. Isolation of BMSCs and BMSC-Derived EVs

**Figure 1:**
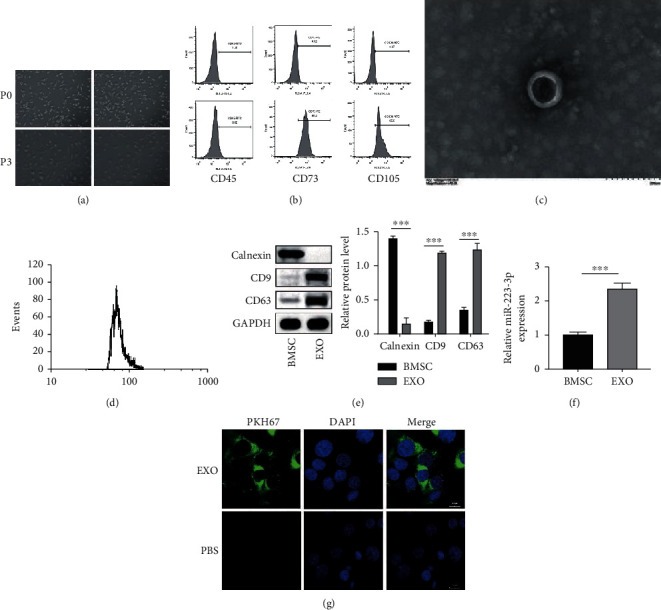
Isolation of BMSCs and BMSC-derived EVs. (a) The morphology of the BMSCs was examined under a light microscope. (b) Flow cytometry was used for identifying the BMSC markers. (c) The morphology of the EVs was observed using TEM. (d) The distribution of the exosome diameters was analyzed. (e) The exosome-specific markers were detected using the western blot analysis. (f) The enrichment of miR-223-3p in the EVs was determined using qPCR. (g) The uptake of PKH67-labelled EVs by the TCMK-1 cells was observed under a fluorescence microscope. *N* = 3. ^∗^*P* < 0.05; ^∗∗^*P* < 0.01; ^∗∗∗^*P* < 0.001.

First, the BMSCs were isolated from the mice using the method described in Section 2. The morphology of the isolated BMSCs is illustrated in Figure 1(a). Next, the BMSC markers were identified using flow cytometry. As depicted in Figure 1(b), the isolated BMSCs tested positive for CD73 (98.2%) and CD105 (95.6%) while negative for CD45 (6.5%). This result validated the purity of the BMSCs. Next, the EVs of the isolated BMSC were isolated using ultracentrifugation. The morphology of the isolated EVs was observed using TEM (Figure 1(c)). The distribution of the exosome diameters was also determined. The results revealed that the median diameter of the isolated EVs was 71.25 nm (Figure 1(d)). The results showed that the main component of EVs were exosomes in this study. The exosome-specific markers were detected using western blot analysis. As depicted in Figure 1(e), the expressions of the exosome markers CD9 and CD63 were high in the isolated EVs and weak in the BMSCs. On the contrary, the expression of the ER stress marker Calnexin was not detected in the EVs. The miRNAs were generally enriched in the EVs. Further, the expression of miR-223-3p in the isolated EVs was analyzed. It was revealed that the miR-223-3p expression was higher in the EVs compared to that in the BMSCs, which indicated the enrichment of miR-223-3p in EVs (Figure 1(f)). Moreover, the secreted EVs could be uptaken by cells. The results presented in Figure 1(g) validated that the TCMK-1 cells could uptake the PKH67-labelled EVs, as evidenced by the green fluorescence emission from the TCMK-1 cells. Collectively, these results demonstrated the successful isolation and identification of BMSCs and BMSC-derived EVs.

### 3.2. BMSC-Derived EVs Promoted Mitophagy and Attenuated Inflammasome Activation to Ameliorate RIRI

**Figure 2:**
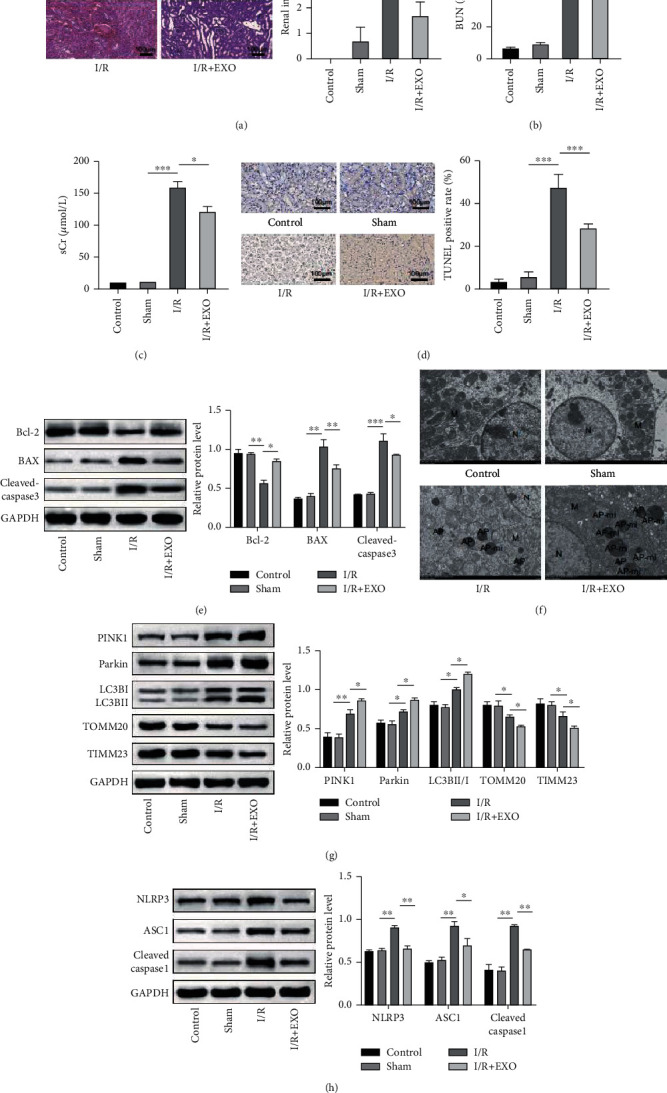
BMSC-derived EVs promoted mitophagy and attenuated inflammasome activation to ameliorate RIRI. Mice were treated with EVs and then subjected to I/R injury. (a) Histological injury was evaluated based on the H&E staining. (b and c) The blood urea nitrogen (BUN) and serum creatinine (sCr) contents were detected using the specific kits. (d) Tissue apoptosis was detected using the TUNEL assay kit. (e) The protein levels of BAX, cleaved caspase-3, and Bcl-2 were confirmed using the western blot analysis. (f) The autophagosomes (APs) and autophagosome-fused mitochondria (AP-mi) were observed using TEM. (g) Expressions of the mitophagy markers were detected using the western blot analysis. (h) The expressions of NLRP3, ASC1, and cleaved caspase 1 were evaluated using the western blot analysis. *n* = 6. ^∗^*P* < 0.05; ^∗∗^*P* < 0.01; ^∗∗∗^*P* < 0.001.

In order to explore the protective effect of the BMSC-derived EVs against RIRI, a mouse model of RIRI was established in the present study by injecting EVs into the mice. As revealed by the H&E staining results, the histological injury was evident in the I/R group compared to the Sham group, although the injection of EVs led to a substantial decrease in the histological injury (Figure 2(a)). Moreover, the renal injury markers blood urea nitrogen (BUN) and serum creatinine (sCr) were also detected. As depicted in Figure 2(b) and Figure 2(c), the BUN and sCr contents were dramatically elevated in the I/R group. However, the injection of EVs sharply attenuated the BUN and sCr contents. Next, the level of apoptosis in the renal tissue was assessed. The results revealed that the exosome treatment notably decreased the I/R-induced TUNEL positive cells (Figure 2(d)). Consistent with this result, I/R was observed to induce the expression of the proapoptotic markers BAX and cleaved caspase-3 while it decreased the expression of the antiapoptotic marker Bcl-2. Interestingly, the exosome treatment reversed this expression pattern (Figure 2(e)). Mitophagy is generally activated in the I/R condition to exert a protective effect [8]. Therefore, the mitochondria in each group were observed next using TEM. As depicted in Figure 2(f), autophagosomes (APs) and autophagosome-fused mitochondria (AP-mi) were observed in the I/R group compared to the control group or Sham group. Importantly, a greater number of AP-mi were presented in the exosome-treated group. Furthermore, the expression of mitophagy markers was analyzed to evaluate mitophagy. It was revealed that I/R induced the expressions of PINK1 and Parkin, enhanced the LC3BII/LC3BI ratio, and decreased the expressions of TOMM20 and TIMM23, which indicated the activation of mitophagy. Moreover, the exosome treatment was observed to further elevate the expressions of PINK1 and Parkin and the LC3BII/LC3BI ratio while it further attenuated the expressions of TOMM20 and TIMM23. The inflammasome is reported to be involved in the I/R condition to induce an excessive inflammatory response. Therefore, the activation of inflammasome was assessed in the present study. It was revealed that exosome treatment could attenuate the I/R-induced expressions of NLRP3, ASC1, and cleaved caspase 1, which indicated the suppression of I/R-induced inflammasome activation. Collectively, these results demonstrated that exosome treatment suppressed apoptosis and inflammasome activation, promoted mitophagy, and alleviated renal injury in the I/R mouse model.

### 3.3. BMSC-Derived EVs Suppressed Apoptosis and Inflammasome Activation and Promoted Mitophagy in the H/R-Induced TCMK-1 Cells

**Figure 3:**
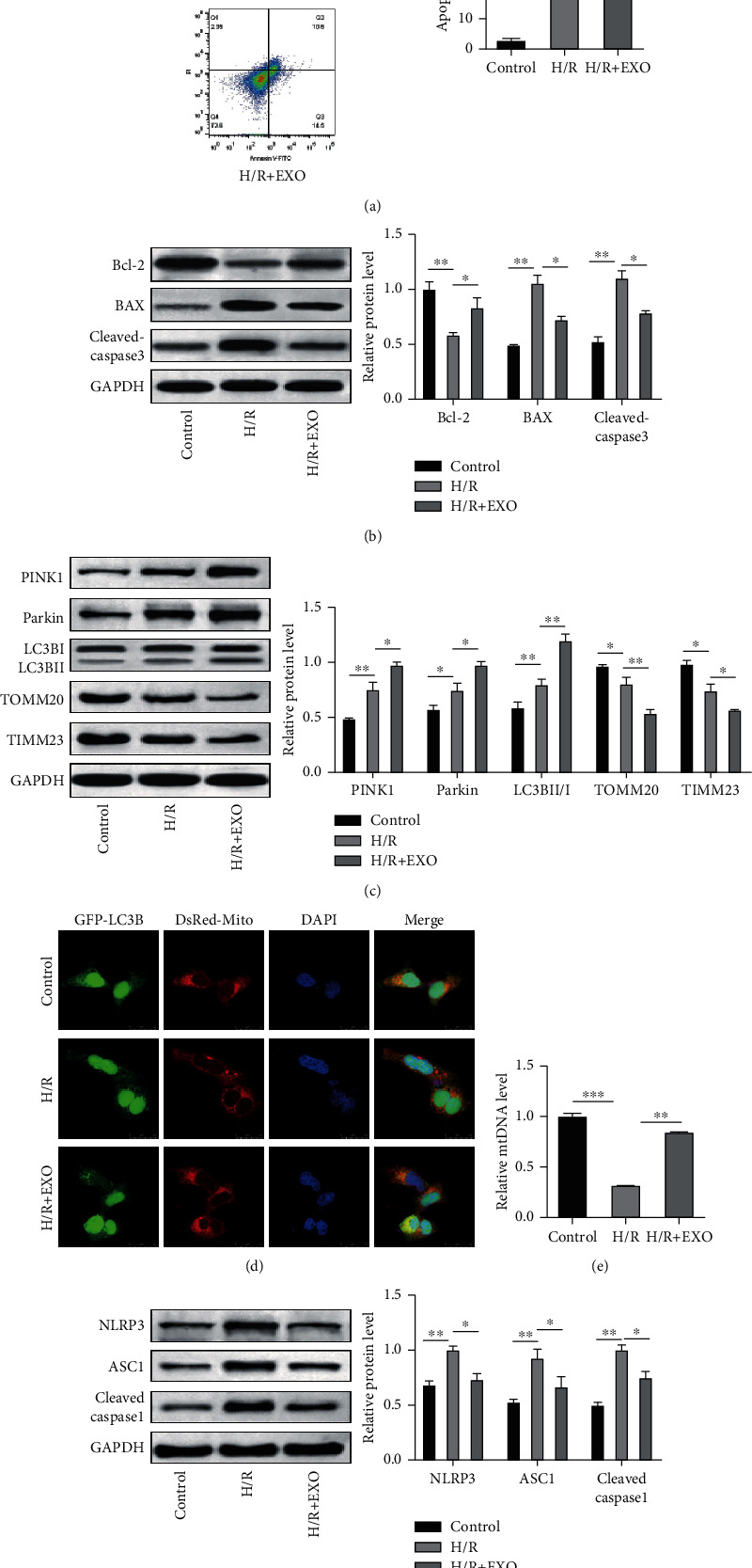
BMSC-derived EVs suppressed apoptosis and inflammasome activation and promoted mitophagy in H/R-induced TCMK-1 cells. TCMK-1 cells were subjected to H/R stimulation and exosome treatment. (a) The H/R-induced cell apoptosis was determined using flow cytometry. (b and e) The protein levels of BAX, cleaved caspase-3, and Bcl-2 were confirmed using the western blot analysis. (c) Expressions of the mitophagy markers were detected using the western blot analysis. (d) Mitophagosome formation was assessed based on the colocalization of the autophagy marker GFP-LC3B and the mitochondria marker Mito Tracker (DsRed-Mito). (e) The levels of mitochondrial DNA (mtDNA) were determined using qPCR. (f) The expressions of NLRP3, ASC1, and cleaved caspase 1 were evaluated using the western blot analysis. *N* = 3. ^∗^*P* < 0.05; ^∗∗^*P* < 0.01; ^∗∗∗^*P* < 0.001.

In order to verify the protective role of the BMSC-derived EVs *in vitro*, the TCMK-1 cells were subjected to H/R stimulation to mimic RIRI *in vitro*. As depicted in Figure 3(a), H/R stimulation induced cell apoptosis while the exosome treatment substantially attenuated the proportion of the apoptotic cells. This result was supported by the expressions of the apoptosis-related proteins (Bcl-2, BAX, and cleaved caspase 3), as depicted in Figure 3(b). The western blot analysis also revealed that H/R enhanced the expressions of PINK1 and Parkin and the LC3BII/LC3BI ratio and decreased the expressions of TOMM20 and TIMM23. Interestingly, exosome treatment further promoted this expression pattern, which indicated enhanced mitophagy in the TCMK-1 cells (Figure 3(c)). The mitophagosome formation was assessed based on the colocalization of the autophagy marker GFP-LC3B and the mitochondria marker Mito Tracker (DsRed-Mito). As depicted in Figure 3(d), colocalization was observed in the H/R-simulated TCMK-1 cells, which indicated mitophagosome formation. Moreover, this colocalization was enhanced by the exosome treatment. The levels of mitochondrial DNA (mtDNA) were also observed to be reduced by the H/R stimulation, while the exosome treatment recovered the levels of mtDNA (Figure 3(e)). Similar to the findings of the *in vivo* experiments, the expressions of NLRP3, ASC1, and cleaved caspase 1 were elevated upon H/R simulation while these expressions were decreased by the exosome treatment, which indicated that the H/R-induced inflammasome activation was impaired upon the exosome treatment (Figure 3(f)). Collectively, these results confirmed the protective role of EVs in an *in vitro* IRIR model.

### 3.4. Exosome-Delivered miR-223-3p Mediated the Protective Effect of EVs in RIRI

**Figure 4:**
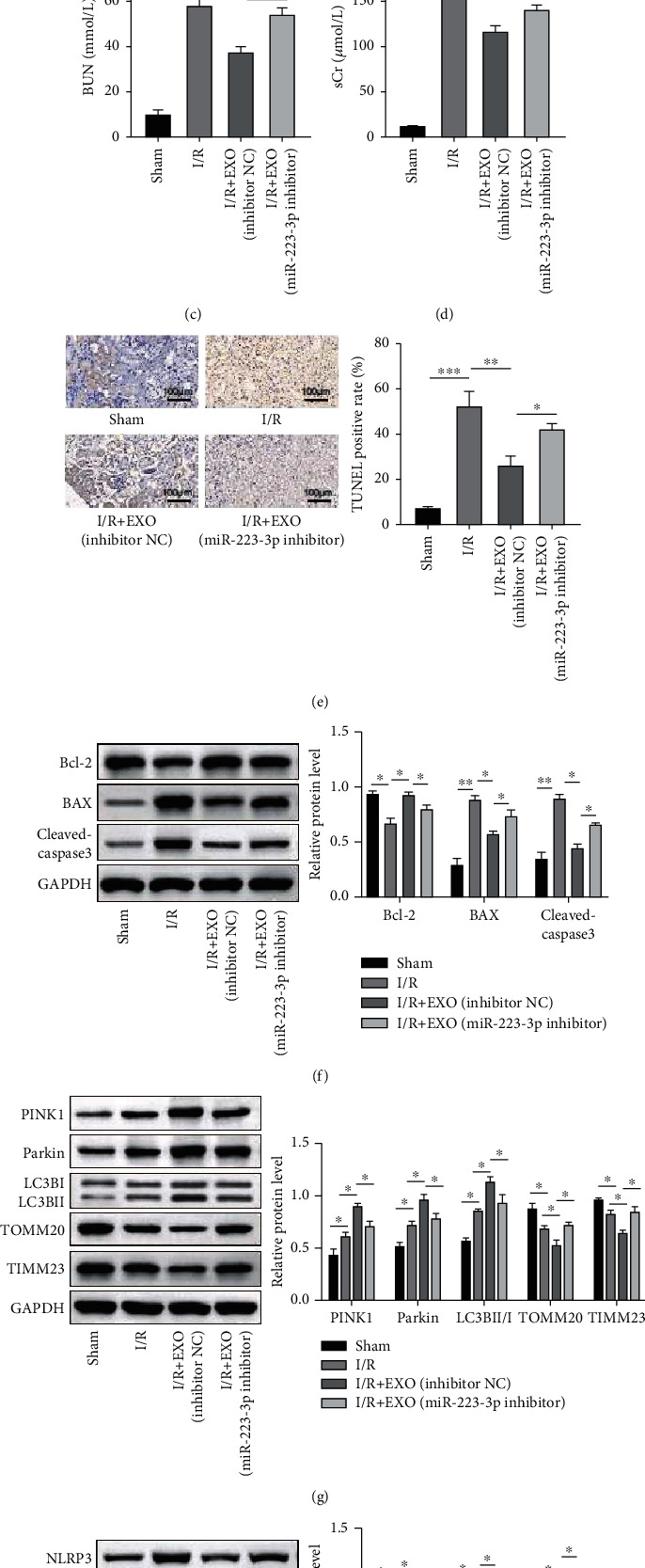
Exosome-delivered miR-223-3p mediated the protective effect of exosome in RIRI. Mice were treated with miR-233-3p-depleted EVs and then subjected to I/R injury. (a) The expression of miR-223-3p was determined using qPCR. (b) Histological injury was evaluated based on H&E staining. (c and d) The blood urea nitrogen (BUN) and serum creatinine (sCr) contents were detected using the specific kits. (e) Tissue apoptosis was detected using the TUNEL assay kit. (f) The protein levels of BAX, cleaved caspase-3, and Bcl-2 were confirmed using the western blot analysis. (g) Expressions of the mitophagy markers were detected using the western blot analysis. (h) The expressions of NLRP3, ASC1, and cleaved caspase 1 were evaluated using the western blot analysis. *n* = 6. ^∗^*P* < 0.05; ^∗∗^*P* < 0.01; ^∗∗∗^*P* < 0.001.

The observed protective effect of EVs against I/R injury was confirmed via miRNA delivery [25]. It is reported that miR-223-3p is involved in the functioning of EVs in cerebral ischemia/reperfusion injury [24]. Therefore, whether miR-223-3p participated in the functioning of EVs in RIRI was investigated in the present study. Silencing of miR-223-3p was achieved using the miR-223-3p inhibitor. The qPCR results validated that miR-223-3p expression was substantially repressed by the miR-223-3p inhibitor in the BMSCs and BMSC-derived EVs (Figure 4(a)). H&E staining revealed that EVs from the inhibitor NC-transfected BMSCs ameliorated the I/R-induced tissue injury. However, miR-223-3p-depleted exosome treatment recovered the tissue injury score (Figure 4(b)). Similarly, I/R-induced BUN and sCr contents were dramatically decreased upon exosome treatment, while treatment with miR-223-3p-depleted EVs restored the BUN and sCr contents (Figure 4(c) and Figure 4(d)). The TUNEL assay and western blot analysis also revealed that the miR-223-3p-depleted EVs partly reversed the protective effect of EVs on cell apoptosis (Figure 4(e) and Figure 4(f)). In regard to mitophagy, it was observed that the EVs from the inhibitor NC-transfected cells promoted the mitophagy further in the renal tissue while the miR-223-3p-depleted EVs partly attenuated mitophagy, as evidenced by the protein expression pattern depicted in Figure 4(g). Importantly, miR-223-3p depletion in EVs also elevated the levels of NLRP3, ASC1, and cleaved caspase 1, which indicated the activation of inflammasome (Figure 4(h)). Collectively, these results suggested that exosome-delivered miR-223-3p mediated the protective effect of EVs in RIRI.

### 3.5. Exosome-Delivered miR-223-3p Mediated the Protective Effect of EVs in H/R-Stimulated TCMK-1 Cells

**Figure 5:**
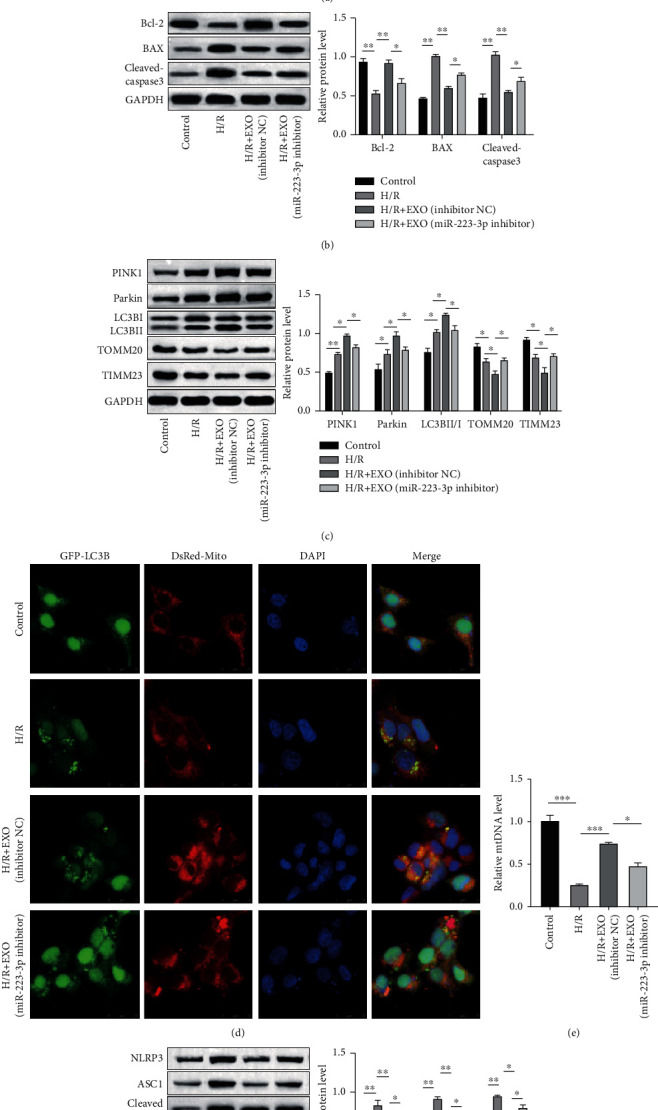
Exosome-delivered miR-223-3p mediated the protective effect of EVs in H/R-stimulated TCMK-1 cells. TCMK-1 cells were subjected to H/R stimulation and miR-223-3p-depleted exosome treatment. (a) The H/R-induced cell apoptosis was determined using flow cytometry. (b) The protein levels of BAX, cleaved caspase-3, and Bcl-2 were confirmed by performing the western blot analysis. (c) Expressions of the mitophagy markers were detected using the western blot analysis. (d) Mitophagosome formation was assessed based on the colocalization of the autophagy marker GFP-LC3B and the mitochondria marker Mito Tracker (DsRed-Mito). (e) The levels of mitochondrial DNA (mtDNA) were determined through qPCR. (f) The expressions of NLRP3, ASC1, and cleaved caspase 1 were evaluated using the western blot analysis. *N* = 3. ^∗^*P* < 0.05; ^∗∗^*P* < 0.01; ^∗∗∗^*P* < 0.001.

As described above, it was validated that miR-223-3p was involved in the protective effect of EVs in RIRI. In order to verify this role of miR-223-3p *in vitro* as well, TCMK-1 cells were subjected to H/R stimulation followed by treatment with miR-223-3p-depleted EVs. The Annexin V/PI staining and western blot analysis results suggested that miR-223-3p inhibition weakened the protective effect of EVs on cell apoptosis (Figure 5(a) and Figure 5(b)). Moreover, the TCMK-1 cells treated with miR-223-3p-depleted EVs exhibited a lower degree of mitophagy, as evidenced by the protein expression pattern and lower mitophagosome formation (Figure 5(c) and Figure 5(d)). In addition, the TCMK-1 cells treated with miR-223-3p-depleted EVs presented lower levels of mtDNA compared to the cells treated with inhibitor NC EVs (Figure 5(e)). As expected, enhanced inflammasome activation was observed in the TCMK-1 cells treated with miR-223-3p-depleted EVs compared to those treated with the inhibitor NC exosome (Figure 5(f)). Collectively, these *in vitro* experimental results validated the finding of the *in vivo* study that the exosome-delivered miR-223-3p mediated the protective effect of EVs.

### 3.6. miR-223-3p Targeted NLRP3

**Figure 6:**
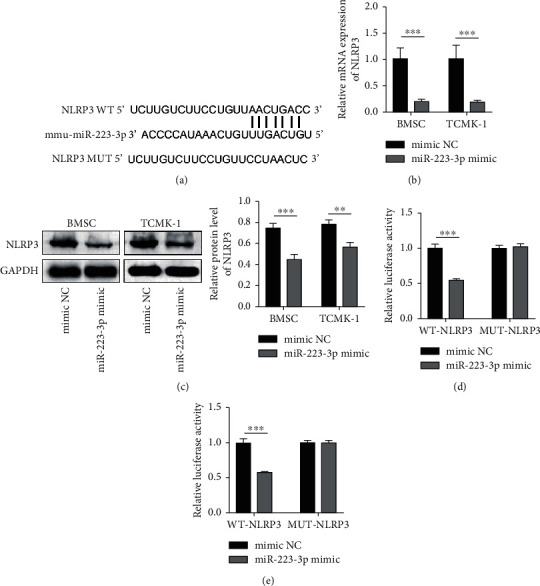
miR-223-3p targeted NLRP3. (a) A potential binding site of miR-223-3p is illustrated. (b and c) The expression of NLRP3 was determined at the mRNA and protein levels through qPCR and western blot analysis, respectively. (d and e) The dual-luciferase assay was used for validating the direct binding of miR-223-3p to NLRP3. *N* = 3. ^∗^*P* < 0.05; ^∗∗^*P* < 0.01; ^∗∗∗^*P* < 0.001.

In order to investigate the mechanism underlying the role of miR-223-3p in RIRI, the downstream targets of miR-223-3p were first identified using TargetScan 7.2 (http://www.targetscan.org/vert_72/). A potential binding site of miR-223-3p was located in the 3′UTR of the NLRP3 mRNA (Figure 6(a)). Next, miR-223-3p was overexpressed in BMSCs and TCMK-1 cells, and the expression of NLRP3 in these cells was determined. The results revealed that miR-223-3p overexpression notably repressed the expression of NLRP3 at both mRNA and protein levels (Figure 6(b) and Figure 6(c)). In order to validate the direct binding of miR-223-3p to the NLRP3 mRNA, the dual-luciferase assay was performed. The assay results revealed that the relative luciferase activity was suppressed when the miR-223-3p mimic was cotransfected with the wild-type binding sequence-containing reporters. However, the relative luciferase activity was not significantly altered when the miR-223-3p mimic was cotransfected with the mutated binding sequence-containing reporters (Figure 6(d) and Figure 6(e)). Collectively, these results demonstrated that miR-223-3p targeted NLRP3, thereby negatively regulating the NLRP3 expression.

### 3.7. miR-223-3p Suppressed Apoptosis and Inflammasome Activation and Promoted Mitophagy in the H/R-Induced TCMK-1 Cells by Targeting NLRP3

**Figure 7:**
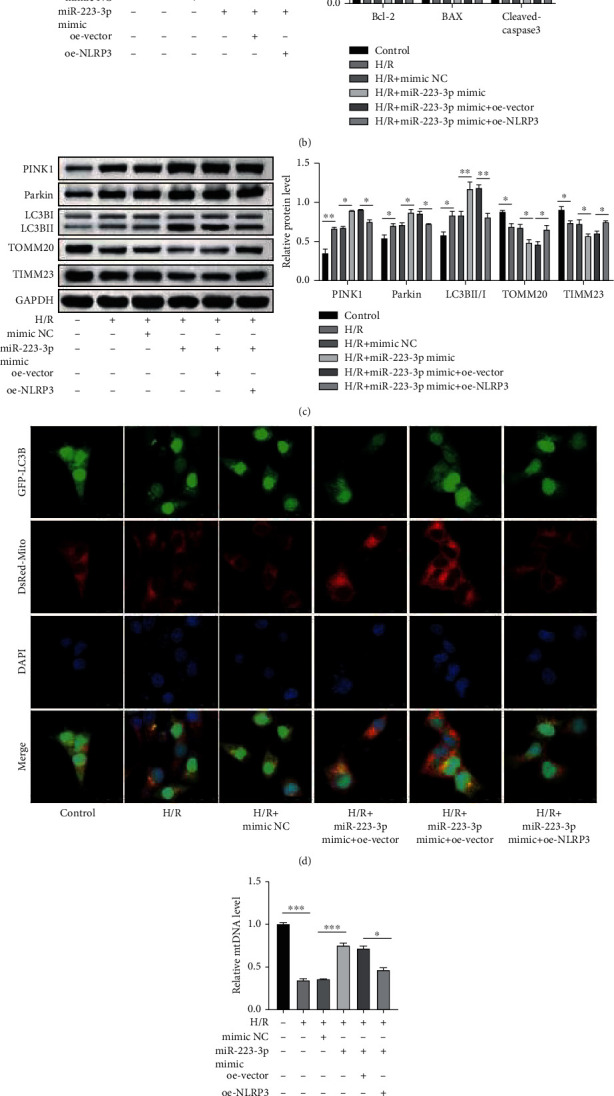
miR-223-3p suppressed apoptosis and inflammasome activation and promoted mitophagy in H/R-induced TCMK-1 cells by targeting NLRP3. TCMK-1 cells were subjected to H/R stimulation and miR-223-3p-depleted exosome treatment. (a) H/R-induced cell apoptosis was determined through flow cytometry. (b) The protein levels of BAX, cleaved caspase-3, and Bcl-2 were confirmed by performing the western blot analysis. (c) Expressions of the mitophagy markers were detected using western blot analysis. (d) Mitophagosome formation was assessed based on the colocalization of the autophagy marker GFP-LC3B and the mitochondria marker Mito Tracker (DsRed-Mito). (e) The levels of mitochondrial DNA (mtDNA) were determined through qPCR. (f) The expressions of NLRP3, ASC1, and cleaved caspase 1 were determined using the western blot analysis. *N* = 3. ^∗^*P* < 0.05; ^∗∗^*P* < 0.01; ^∗∗∗^*P* < 0.001.

The next step was to investigate whether miR-223-3p exerted a protective effect in H/R-induced TCMK-1 cells and whether this protective effect was mediated by the NLRP3 suppression. This investigation was performed by miR-223-3p and/or NLRP3 overexpression in TCMK-1 cells. As depicted in Figure 7(a), miR-223-3p overexpression attenuated the H/R-induced apoptosis, while NLRP3 overexpression partly compromised this effect. The western blot analysis results also suggested that miR-223-3p overexpression elevated Bcl-2 and decreased the BAX and cleaved caspase 3 levels while the NLRP3 overexpression partly reversed this expression pattern (Figure 7(b)). NLRP3 is reported as a negative regulator of mitophagy [26]. Interestingly, the findings of the present study also suggested that miR-223-3p promoted mitophagy in H/R-stimulated TCMK-1 cells, as evidenced by the protein expression pattern and the confocal microscopy images. However, the overexpression of NLRP3 attenuated mitophagy in H/R-stimulated TCMK-1 cells (Figures 7(c) and 7(d)). Similarly, NLRP3 overexpression also impaired the effect of miR-223-3p mimic on the elevation of mtDNA levels (Figure 7(e)). Finally, as expected, miR-223-3p impaired the inflammasome activation induced by H/R stimulation. However, the overexpression of NLRP3 reactivated the inflammasome (Figure 7(f)). Collectively, these results validated that miR-223-3p suppressed apoptosis and inflammasome activation and promoted mitophagy in the H/R-induced TCMK-1 cells by targeting NLRP3.

## 4. Discussion

In the present study, the beneficial role of BMSC-derived EVs in RIRI-caused kidney injury was validated *in vitro* and *in vivo*. The treatment with BMSC-derived EVs was observed to attenuate apoptosis and inflammasome activation and promoted mitophagy in RIRI. The results of the mechanism study revealed that the exosome-delivered miR-223-3p mediated the protective role of BMSC-derived EVs by targeting NLRP3, thereby inhibiting the activation of the inflammasome and eliciting protective mitophagy.

Previous studies have reported the mediation of several miRNAs in the protective role of EVs in RIRI. For instance, Zhu et al. validated that the EVs from BMSCs protected against RIRI by delivering miR-199a-3p [27]. Li et al. reported that exosome-derived miR-146a-5p ameliorated RIRI by targeting IRAK1 [28]. The delivery of miR-216a-5p from EVs was confirmed to reduce RIRI in another study [29]. According to another report, miR-223-3p exhibited a protective role in myocardial ischemia/reperfusion [23]. In addition, miR-223-3p is reportedly involved in the functional role of EVs in cerebral ischemia/reperfusion [24]. The present study, however, was the first to confirm that BMSC-derived EVs alleviated RIRI by delivering miR-223-3p. The results revealed that the depletion of miR-223-3p in EVs sharply weakened the protective effect of the exosome treatment against RIRI. In addition, the results verified that miR-223-3p overexpression exerted a protective effect similar to that of exosome treatment in the *in vitro* RIRI model.

In recent years, mitophagy has been demonstrated as a protective process in renal ischemia/reperfusion injury [7, 8]. Besides RIRI, mitophagy is also reported as a novel therapeutic target in cerebral ischemia [9] and myocardial ischemia [30]. Moreover, it is suggested that EVs might alleviate mitochondrial dysfunction [31, 32]. However, there is no evidence to support that EVs affect mitophagy directly. In the present study, mitophagy was elicited upon I/R or H/R treatment, which is consistent with the findings of previous studies [8]. Importantly, treatment with EVs further promoted the activation of mitophagy, indicating the protective effect of EVs. Moreover, miR-223-3p was observed to exhibit effects similar to those exhibited by exosome treatment, i.e., enhancement of mitophagy. These findings unveiled a novel mechanism through which EVs and the exosome-delivered miRNA alleviated RIRI. In general, mitophagy has been demonstrated to be beneficial against ischemia/reperfusion injury. However, it is noteworthy that the role of autophagy in this scenario remains debatable. While FGF10 is reported to ameliorate RIRI via inhibition of autophagy [33], trehalose reportedly attenuates RIRI by activating autophagy [34].

NLRP3 inflammasome is a large intracellular complex comprising multiple proteins. This complex is essential for the activation of the inflammatory response and programmed inflammatory cell death (pyroptosis) [35]. It is well-recognized that inflammasome activation is enhanced in RIRI and, therefore, inhibition of inflammasome activation is an effective approach to protect against RIRI [36, 37]. In the present study, it was observed that the exosome treatment and the overexpression of miR-223-3p substantially attenuated the activation of the NLRP3 inflammasome. This was consistent with the finding of Dai et al., who reported that the exosome-delivered miR-148a alleviated myocardial ischemia/reperfusion injury by abrogating the TXNIP-mediated activation of the NLRP3 inflammasome [38]. The bioinformatic analysis followed by the experimental validation in the present study revealed that miR-223-3p directly targeted and downregulated NLRP3, thereby abrogating the inflammasome activation. This was consistent with the previous studies that reported the negative regulation of inflammasome activation by miR-223-3p via NLRP3 targeting in other diseases [39, 40].

Interestingly, while the simultaneous overexpression of NRLP3 and miR-223-3p restored the activation of the inflammasome and apoptosis, it also repressed the elicited mitophagy, which indicated a regulatory role of the NLRP3 inflammasome in mitophagy. This finding is consistent with several previous reports. As reported by Lin et al., the inhibition of the NLRP3 inflammasome decreased apoptosis in contrast-induced AKI via the upregulation of HIF-1*α* and BNIP3-mediated mitophagy [26]. Wu et al. reported that NLRP3 knockdown was protected against intermittent hypoxia-induced injury by enhancing the PINK1-Parkin-mediated mitophagy [41]. Interestingly, it is reported by several studies that mitophagy negatively regulates NLRP3 [42–44]. These studies, along with the present one, confirm the reciprocal regulation of mitophagy and the NLRP3 inflammasome, which could be involved in the control of intracellular homeostasis. Although the results of the present study confirmed that EVs and miR-223-3p regulated mitophagy by impairing the activation of the NLRP3 inflammasome, further investigation is required to reveal whether the EVs and miR-223-3p regulate mitophagy directly or indirectly.

In conclusion, the protective roles of MSC-derived EVs with ~70 nm diameter and EVs-delivered miR-223-3p in RIRI were validated. Exogenous miR-223-3p directly targeted NLRP3 to attenuate inflammasome activation, thereby promoting mitophagy. The findings of the present study would contribute to understanding the protective regulatory role of EVs in RIRI.

## Data Availability

The original contributions presented in the study are included in the article, further inquiries can be directed to the corresponding author.

## Abbreviation

(AKI): Acute kidney injury
(RIRI): Renal ischemia/reperfusion injury
(BNIP3): BCL2 interacting protein 3
(FUNDC1): FUN14 domain-containing 1
(HIF-1*α*): Hypoxia-inducible factor 1*α*
(MSC): Mesenchymal stem cells
(miRNA): microRNA
(NLRP3): NLR family pyrin domain-containing 3
(BUN): Blood urea nitrogen
(sCr): Serum creatinine
(FGF10): Fibroblast growth factor 10
(TXNIP): Thioredoxin-interacting protein
(CCR2): C-C motif chemokine receptor-2.
